# Assessing the Digital Advancement of Public Health Systems Using Indicators Published in Gray Literature: Narrative Review

**DOI:** 10.2196/63031

**Published:** 2024-11-20

**Authors:** Laura Maaß, Manuel Badino, Ihoghosa Iyamu, Felix Holl

**Affiliations:** 1 SOCIUM Research Center on Inequality and Social Policy University of Bremen Bremen Germany; 2 Leibniz ScienceCampus Digital Public Health Bremen Bremen Germany; 3 Digital Health and Artificial Intelligence Section European Public Health Association (EUPHA) Utrecht Netherlands; 4 School of Public Health National University of Córdoba Córdoba Argentina; 5 Public Health and Epidemiology Research Group School of Medicine and Health Sciences University of Alcalá Alcalá de Henares, Madrid Spain; 6 School of Population and Public Health University of British Columbia Vancouver, BC Canada; 7 BC Centre for Disease Control Vancouver, BC Canada; 8 DigiHealth Institute Neu-Ulm University of Applied Sciences Neu-Ulm Germany

**Keywords:** digital public health, health system, indicator, interdisciplinary, information and communications technology, maturity assessment, readiness assessment, narrative review, gray literature, digital health, mobile phone

## Abstract

**Background:**

Revealing the full potential of digital public health (DiPH) systems requires a wide-ranging tool to assess their maturity and readiness for emerging technologies. Although a variety of indices exist to assess digital health systems, questions arise about the inclusion of indicators of information and communications technology maturity and readiness, digital (health) literacy, and interest in DiPH tools by the society and workforce, as well as the maturity of the legal framework and the readiness of digitalized health systems. Existing tools frequently target one of these domains while overlooking the others. In addition, no review has yet holistically investigated the available national DiPH system maturity and readiness indicators using a multidisciplinary lens.

**Objective:**

We used a narrative review to map the landscape of DiPH system maturity and readiness indicators published in the gray literature.

**Methods:**

As original indicators were not published in scientific databases, we applied predefined search strings to the DuckDuckGo and Google search engines for 11 countries from all continents that had reached level 4 of 5 in the latest Global Digital Health Monitor evaluation. In addition, we searched the literature published by 19 international organizations for maturity and readiness indicators concerning DiPH.

**Results:**

Of the 1484 identified references, 137 were included, and they yielded 15,806 indicators. We deemed 286 indicators from 90 references relevant for DiPH system maturity and readiness assessments. The majority of these indicators (133/286, 46.5%) had legal relevance (targeting big data and artificial intelligence regulation, cybersecurity, national DiPH strategies, or health data governance), and the smallest number of indicators (37/286, 12.9%) were related to social domains (focusing on internet use and access, digital literacy and digital health literacy, or the use of DiPH tools, smartphones, and computers). Another 14.3% (41/286) of indicators analyzed the information and communications technology infrastructure (such as workforce, electricity, internet, and smartphone availability or interoperability standards). The remaining 26.2% (75/286) of indicators described the degree to which DiPH was applied (including health data architecture, storage, and access; the implementation of DiPH interventions; or the existence of interventions promoting health literacy and digital inclusion).

**Conclusions:**

Our work is the first to conduct a multidisciplinary analysis of the gray literature on DiPH maturity and readiness assessments. Although new methods for systematically researching gray literature are needed, our study holds the potential to develop more comprehensive tools for DiPH system assessments. We contributed toward a more holistic understanding of DiPH. Further examination is required to analyze the suitability and applicability of all identified indicators in diverse health care settings. By developing a standardized method to assess DiPH system maturity and readiness, we aim to foster informed decision-making among health care planners and practitioners to improve resource distribution and continue to drive innovation in health care delivery.

## Introduction

### Background

The digital transformation of systems throughout society, particularly within health systems, has substantial implications for public health [1,2]. Practitioners and researchers agree that the digital transformation of health care systems can potentially shift the nature of health services from curative to preventive services that promote equity and optimal health outcomes within communities [3,4]. This shift, facilitated by the digital transformation of public health systems, is an opportunity to address long-standing public health challenges such as rising health care costs, aging populations, and human resource shortages [5,6]. Public health systems include not only the traditional health care delivery system but also government-run public health agencies, academia, and additional sectors that are engaged in health activities such as social care or schools. As such, they broaden the clinical understanding of health systems [7]. However, national governments, nongovernmental organizations, and intergovernmental organizations, including the World Health Organization, have developed comprehensive strategies to facilitate the digital transformation of health systems targeting clinical health care [8,9]. None of these is capable of addressing the more holistic public health systems that go beyond health care. Instead, these strategies aim to reorganize health services and operations to deeply integrate digital technologies at every level of health care delivery [10]. While these strategies have broadly explored the digital transformation of health systems, there is increasing recognition of the opportunities and challenges of this process, specifically in terms of public health services and functions. The recognition has led to the emergence of digital public health (DiPH) as a critical focus area [10-12].

### The Need for DiPH

Building on the concept of public health as the “science and art of preventing disease, prolonging life and promoting, protecting and improving health through the organized efforts of society” [13], DiPH considers digital transformation as “an asset community has to [fulfill] its [public health] aims and mission” [4]. It leverages digital technologies to achieve public health system goals of quality, accessibility, efficiency, and health care equity with an impact on the health of the population [4,14]. Consequently, DiPH tools are public goods that are accessible and beneficial to different social groups without charges, including health-specific hardware and software applications for recording, monitoring, evaluating, and intervening to optimize specific health parameters at a population, community, and global health level [15]. It addresses the increasing role of digital technologies in health promotion and protection and the role of DiPH in managing the public health risks of the widespread uptake of digital technologies [16,17].

Widespread digital transformation within public health systems leads to inherent technological and health care complexities that must be reconciled with the complex interplay of intra- and interjurisdictional sociopolitical, economic, and technical contexts that influence public health outcomes. As such, digitalization processes within public health systems require involving various stakeholders within and outside the health sector who must navigate the ethical, legal, financial, regulatory, and infrastructure issues at scale to effectively leverage digital transformation to ensure the attainment of public health goals [1,18]. Consequently, incorporating digital transformation strategies as a priority in the agendas of national governments and international organizations needs to involve heterogenic perspectives that only DiPH can offer, given the rather limited perspectives on clinical health care in digital health or telemedicine.

### Current Status of Digital Maturity and Readiness Measurement Approaches

To effectively consolidate digital transformation strategies for DiPH, we must understand how digital technologies are used within public health systems to deliver high-quality services and the capacity of such systems to adapt and integrate new information and communications technologies (ICTs). For these assessments, digital maturity or readiness can be evaluated. Both concepts are often used interchangeably in the literature. However, it is essential to distinguish between the two concepts. Digital maturity assesses the status quo of digital systems. It describes the current degree to which digital tools are used and how IT systems are connected in the health system as enablers to allow high-quality health care delivery [19-21]. Digital readiness evaluations, on the other hand, analyze the preparedness of systems for anticipated change brought by ICT developments to implement and leverage emerging digital technologies effectively [22]. Consequently, readiness is a multifactorial concept that includes domains such as social and organizational culture, policies, and human resources instead of focusing exclusively on investments in IT equipment or infrastructure [23]. Such assessments can inform the development of roadmaps for the continuous innovation and sustainable integration of DiPH interventions to achieve public health goals [19,24]. Existing indicators explore health systems’ capacity for digital technologies in terms of leadership, workforce capacity, infrastructure, legislation, policy and compliance, standards and interoperability, and the existence of national strategies and investments in these technologies [25-27].

### Gaps in Research and Practice of Maturity and Readiness Assessments

While maturity and readiness assessment tools have been developed at least partially to evaluate the digital transformation of health systems, these indices do not effectively capture the far-reaching nature of DiPH, extending beyond clinical health services to consider social systems and other similar domains that significantly impact public health [15]. Where available, within the public health framework, these indices have only focused on the digital maturity of public health agencies and not that of public health systems within jurisdictions as DiPH requires [28]. Although these indices may embrace similar domains as existing tools, including focusing on having a digital transformation strategy, building employee capacity, and adapting processes and infrastructure for digital transformation, their organizational focus may not necessarily translate to a system perspective relevant to DiPH [29]. Establishing indicators for DiPH systems maturity and readiness (DiPHSMR) can help foster clear jurisdictional objectives for the digital transformation process, create benchmarks for evaluating progress across public health systems, ensure that digital transformation goals are realistic, and ensure continued engagement of local and national stakeholders in facilitating public health goals through this process [19].

### Study Aim and Objective

It is necessary to conceptualize, operationalize, and define indicators to measure DiPH systems maturity and readiness. Measuring this though available, well-defined indicators will support policy makers and researchers in measuring the progress of digital transformation and facilitate the identification of shortcomings within public health systems [20,21]. Therefore, this study aims to identify published and validated indicators suitable for measuring DiPH systems maturity and readiness through a multidisciplinary narrative review. With this approach, we strive to complement the results of our previous Delphi study [15] and integrate them into the current canon of DiPH systems maturity and readiness analysis.

## Methods

### Search Strategy

We conducted a narrative review of gray literature on the indicators for DiPH systems maturity and readiness. Our search strategy was collaboratively developed with librarians specialized in health, economics, political sciences, ICTs, and public health. The strategy focused entirely on the gray literature, given our collective conclusion that indicators of interest were more likely to be published as organizational reports or in repositories such as the indicator metadata registry list by the Global Health Observatory [30] rather than as peer-reviewed articles, which would be indexed in more traditional databases. Mahood et al [31] similarly describe the importance of including gray literature for reviews on such practical topics. Our search strategy explored concepts highlighted in our previous Delphi study [15], identifying potential indicators for measuring national DiPH maturity. The search terms were related to the four DiPH overarching domains:

1. The ICT requirements for nationwide roll out and usability of DiPH tools (ICT)
2. The legal framework and political support for regulating DiPH tools (legal)
3. The public’s and workforce’s willingness and capability to use DiPH tools (social)
4. The degree of DiPH tool and service implementation in the health system (application)

### Literature Search

We used 15 maturity measurement tools from the Delphi study [15] that were previously identified and had included at least one developed indicator to decide on a suitable platform for the literature search. We aimed to identify these references by using predefined search terms and search strings (Textbox 1). The complete search, including terms, date, and results, is documented in Multimedia Appendix 1 (pages 5 and 6). We piloted the search strategy with 3 search engines (DuckDuckGo, Google, and Google Scholar) and 3 scientific databases (PubMed, Web of Science, and IEEE Xplore). However, only DuckDuckGo and Google listed all the targeted references and were included as platforms for our search. The scientific databases did not contain any of the references as primary literature. While we identified some scientific articles that mentioned the maturity tools in the scientific databases or Google Scholar, these were merely secondary references and not the primary resource for the indicators. As we aimed to identify raw indicators directly from their providing bodies, we decided to include only DuckDuckGo and, where needed due to geographical restrictions of using DuckDuckGo, Google in our search. Unlike Google, DuckDuckGo does not collect its users’ personal data and therefore claims to display the same results to every user, thus increasing the chance of reproducibility of our search results [32]. For all searches conducted with Google, a second author reran the searches and compared the first 10 results for each term with the author who had performed the initial examination to assess the neutrality of the result display. This was done for all cases. The searches were conducted from September to December 2023.

**Search terms for DuckDuckGo.**

**Digital health and public health**

- “digital health” index
- health index
- “public health” index
- “mobile health” index
- “electronic health” index
- “healthcare access” index

**Policy and data protection**

- government index
- cybersecurity index

**Information and communications infrastructure**

- digital index
- security digital index
- ICT index
- network index
- connectivity index
- internet index

**Willingness and capability to use digital tools**

- “digital literacy” index
- “digital health literacy” index
- “health literacy” index

For the Google search, we turned off the personal results function; set the language of displayed results to English, German, Portuguese, or Spanish (as at least one author in the team can understand these languages fluently); and changed the geographical result location according to the specific country for which the search was conducted. For DuckDuckGo, we only changed the geographical result location because the other options were unavailable during our search. As neither DuckDuckGo nor Google allow sophisticated search strings, we facilitated the search strategy and applied it to both search engines (Textbox 1), following the study by Godin et al [33].

We applied the search strategy to the 2 largest countries of each continent (from a population perspective) that had reached at least level 4 of the total 5 levels in the 2023 update of the Global Digital Health Monitor (GDHM) [34]. The GDHM is currently the most interdisciplinary and holistic tool to assess national digital health maturity and therefore the most suited orientation tool for our purposes. It tracks and evaluates the nationwide use of digital health tools globally and classifies countries based on 23 indicators into one of the 5 phases of development (where the fifth phase is the most advanced) through seven domains (although data are not always provided for each domain per country): (1) leadership and governance; (2) strategy and investment; (3) legislation, policy, and compliance; (4) workforce; (5) standards and interoperability; (6) infrastructure; and (7) services and applications.

We assumed that the higher the GDHM score, the more relevant digital health and DiPH would be for a country. We further assumed that this would increase the chances that search algorithms would show DiPH-related topics and that we would be more likely to identify DiPH systems maturity and readiness indicators. For the Australian continent, we only included Australia. For Africa, neither Tanzania nor Ethiopia was available as a search location in DuckDuckGo. Consequently, Google was used to search for these 2 countries. Table 1 gives an overview of the selected countries.

To support our findings from the country-based search, we followed the approach by Godin et al [33] and searched the websites of relevant international organizations [35-53]. These organizations were broadly recognized for engaging in at least one of the 4 domains we aimed to address in the project and had previously published indicators or indices on at least one of our defined DiPH domains. Publications from each organization were handsearched for indicators or indices not already identified through the initial country-based search. The identified websites and the search dates were documented for the country and the organization searches. Finally, all authors conducted a handsearch among the reference lists of included publications to identify further indicators.

**Table 1:** Overview of selected countries used for the search [34].

Country	Global Digital Health Monitor dimension									Population (million), n
	Overall	D1^a^	D2^b^	D3^c^	D4^d^	D5^e^	D6^f^	D7^g^		
Canada	5	5	—^h^	5	—	—	5	—	39	
United States	5	5	—	5	—	—	5	—	340	
Brazil	4	5	4	4	4	5	5	5	216	
Argentina	4	4	5	5	4	2	3	2	46	
United Kingdom	5	5	—	4	—	—	5	—	68	
Germany	5	5	—	5	—	—	5	—	83	
Ethiopia	4	4	3	3	5	3	4	3	128	
Tanzania	4	5	4	4	2	5	4	4	67	
India	4	3	—	4	—	—	3	—	1428	
China	5	5	—	4	—	—	4	—	1425	
Australia	5	5	—	5	—	—	5	—	26	

^a^D1: leadership and governance domain.

^b^D2: strategy and investment domain.

^c^D3: legislation, policy, and compliance domain.

^d^D4: workforce domain.

^e^D5: standards and interoperability domain.

^f^D6: infrastructure domain.

^g^D7: services and applications domain.

^h^Not available.

### Screening Process and Eligibility Criteria

Each search term was run by one author per country, leading to 11 searches per search term. Three authors participated in the country search, each searching between 3 and 5 countries (Table 1). The organization search was run by 2 authors individually (LM and MB). The investigations were run from September 26 to December 15, 2023. For the country-based searches, we screened only the first page of Google results (60 results) and the first 3 pages (80 results) for DuckDuckGo. We extracted all references that stated the terms index, indices, indicators, or indicators that had not already been extracted. Textbox 2 displays the screening inclusion and exclusion criteria.

Initially, all identified references were collected in an Excel (version 2019; Microsoft Corporation) sheet. Each author independently decided on an initial inclusion or exclusion of the publications based on our predefined criteria. Following the 4-eye principle, another author (LM and MB) screened all extracted references again for eligibility. Disagreements were resolved by discussion between the author who initially extracted the reference and the author who reviewed it.

**Inclusion and exclusion criteria of references.**

**Inclusion criteria**

- Reference is available in English, Portuguese, Spanish, or German.
- Reference addresses at least one of the digital public health system maturity domains according to the Delphi study by Maaß et al [17].
- Reference lists at least one concrete indicator applicable for measuring one domain of digital public health system maturity.

**Exclusion criteria**

- Reference uses the search term as a synonym for a glossary or registry.
- Search term is a company’s, social media account’s, or stock fund’s name.
- Search term refers to a mathematical or informatics code.
- Reference does not mention specific indicators.
- Reference does not fit the topic according to the Delphi study [15].

### Data Extraction

For each given indicator, provided by included references, we extracted the following information in an Excel file: (1) the reference title and link, (2) the name of the providing organization or authors, (3) the description and definition of the indicator, and (4) the data source of the indicator.

Due to the large number of included references, all indicators were extracted by only one author each. For data quality assurance, the indicators of a random 5% sample of the included references were extracted by another author to check for discrepancies. As this was not the case, we are confident that no selection bias impacted our approach.

Due to the vast number of indicators, we assessed the references for eligibility before eliminating duplications. Two authors followed this approach, and conflicts were resolved by discussion between them. We predefined the following inclusion criteria based on the Delphi study [15], the Digital Integration Index [54], and the #SmartHealthSystems Index [26]: if an indicator was also mentioned in the Delphi study [15], it was automatically included. All other indicators (1) needed to be applicable for assessing at the national system level instead of at the individual people or institution level (such as hospitals), (2) needed to be relevant for at least one thematic cluster in DiPH system maturity assessment, (3) needed to be answered through either official national reports or expert opinions on Likert scales, (4) needed to be transparent in terms of their methodology and a trustworthy data source, (5) must be consistent (so the indicator can also be applied for future assessments), (6) must use up-to-date data to display the current maturity of a national system, and (7) must use data sources that are freely and openly accessible.

The remaining indicators were then assessed by 3 authors for duplications and were combined if needed. A duplicate was defined as an indicator with the same terminology, definition, and data source. We then merged the indicators that measured the same construct. Two authors independently clustered the remaining indicators across the 4 overarching DiPH domains and categorized them into smaller clusters based on targeted topics. Conflicts were resolved by discussion between both authors. Cohen κ values were calculated to measure the strength of agreement between merging decisions [55].

For the final inclusion decision, all authors independently assessed the importance of the remaining indicators based on the predefined inclusion criteria and their expertise in DiPH. Every team member provided their decision on a 4-point Likert scale ranging from 1 (not important) to 4 (very important). Indicators for which at least 75% of all authors voted as 3 to 4 were included, while the other indicators were excluded. The selected indicators were then categorized by 2 authors independently, depending on whether they measured digital maturity, digital readiness, or both (based on the definition given earlier). We chose “both” as the final conclusion, where the authors decided differently. We summarized the identified indicators using a narrative synthesis and descriptive statistics, as appropriate.

## Results

### Descriptive Results of the Screening Clustering Procedure for All Indicators

**Figure 1:**
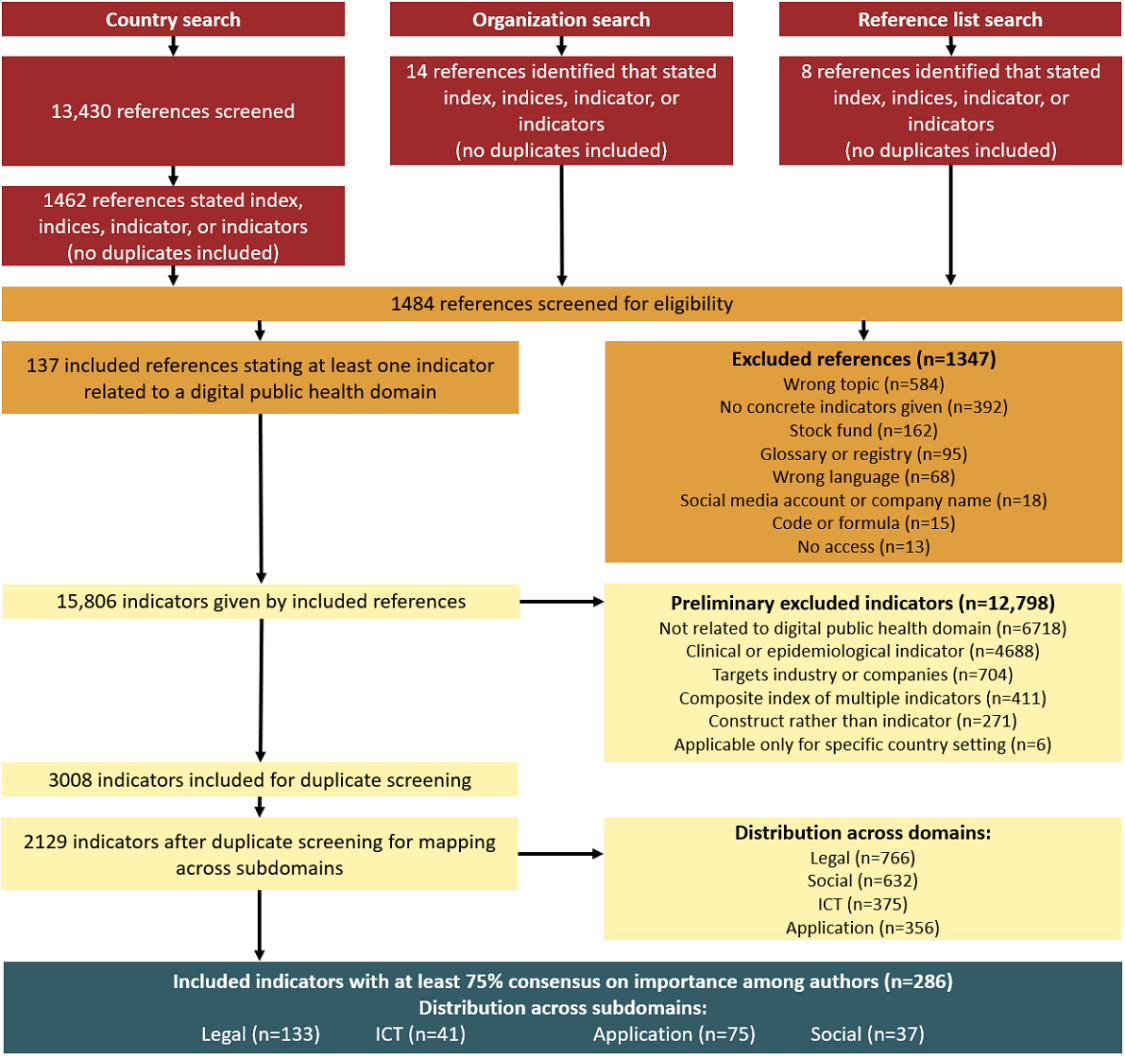
Screening and indicator selection procedure. ICT: information and communications technology.

The overall screening and indicator selection process is displayed in Figure 1. Of the 13,430 screened references from the country-based search, 1462 explicitly named terminology related to indicators (such as index, indices, indicator, or indicators). An additional 14 references were identified through the search on organization websites, and we identified 8 more references through reference list searches among the other publications (red boxes in Figure 1). References naming at least one indicator were screened for eligibility, which resulted in 137 references naming 15,806 indicators (orange boxes in Figure 1).

After assessing the suitability of the indicators and analyzing for duplications, the remaining 2129 indicators were clustered across the 4 overarching domains, with most indicators being distributed to the legal or social domain. Cohen κ values for the overarching domains ranged between 85% and 95%, displaying almost perfect strength of agreement [55]. For the subdomains, κ values ranged from 46% for application to 96% for legal, with an overall average κ value of 77%, displaying the substantial strength of agreement (yellow boxes in Figure 1). However, the clustering process was complicated due to missing indicator descriptions or data sources. In fact, only 39.91% (6308/15,806) of all initial indicators provided a description, and even fewer listed their data sources (4292/15,806, 27.15%).

Eventually, we considered 286 indicators (blue box in Figure 1) as essential to measure national DiPH system maturity and readiness.

### Distribution of Indicators Across Domains

#### Overview

The selected indicators stem from 90 sources [26,27,30,34,56-140]. Six references included at least one indicator per predefined domain [26,27,30,64,68,77]. The majority of indicators were from a legal perspective (133/286, 46.5%), derived from only 27 different references [26,27,30,34,56-78]. The second largest indicator group included those targeting the application of DiPH interventions and services in health care and health promotion settings for which we identified 75 indicators from 29 references [26,27,30,34,56,59,61,66-68,70,71,73-77,79-88]. We also found 41 ICT indicators from 53 references [26,27,30,34,57,62-64,66,68-70,75-77,80,82,88-124], making the social dimension the smallest with 37 indicators provided by 49 references [26,27,30,56,57,61-64,68,73,74,77,81,82, 84-87,91,92,95,98,101-103,110,111,114,115,120,121,124-140].

Regarding maturity or readiness, we categorized 14 indicators as solely describing readiness toward emerging technologies. These included trust in emerging technologies, such as artificial intelligence (AI) from a social perspective [27,73,81], but also having budget plans for new technologies with a population health impact [69] and national policies to promote the implementation of information exchange networks for the later uptake of DiPH interventions [77] with institutions or public bodies being responsible for the execution of such plans [26,77]. By far, the bigger group of indicators was categorized as measuring the system’s current status and, thereby, its digital maturity (8/37, 21% of indicators of the social dimension; 11/41, 26% for ICTs; 42/75, 56% for application; and 49/133, 36.8% for legal indicators). The remaining 162 indicators are applicable for both, maturity and readiness assessment, as they define constructs that are important for the development phase as well as for paving the way for emerging technologies.

All indicators that we deemed essential for an assessment are displayed in Multimedia Appendix 1 (pages 1-4).

#### The Social Dimension of DiPH Maturity

**Figure 2:**
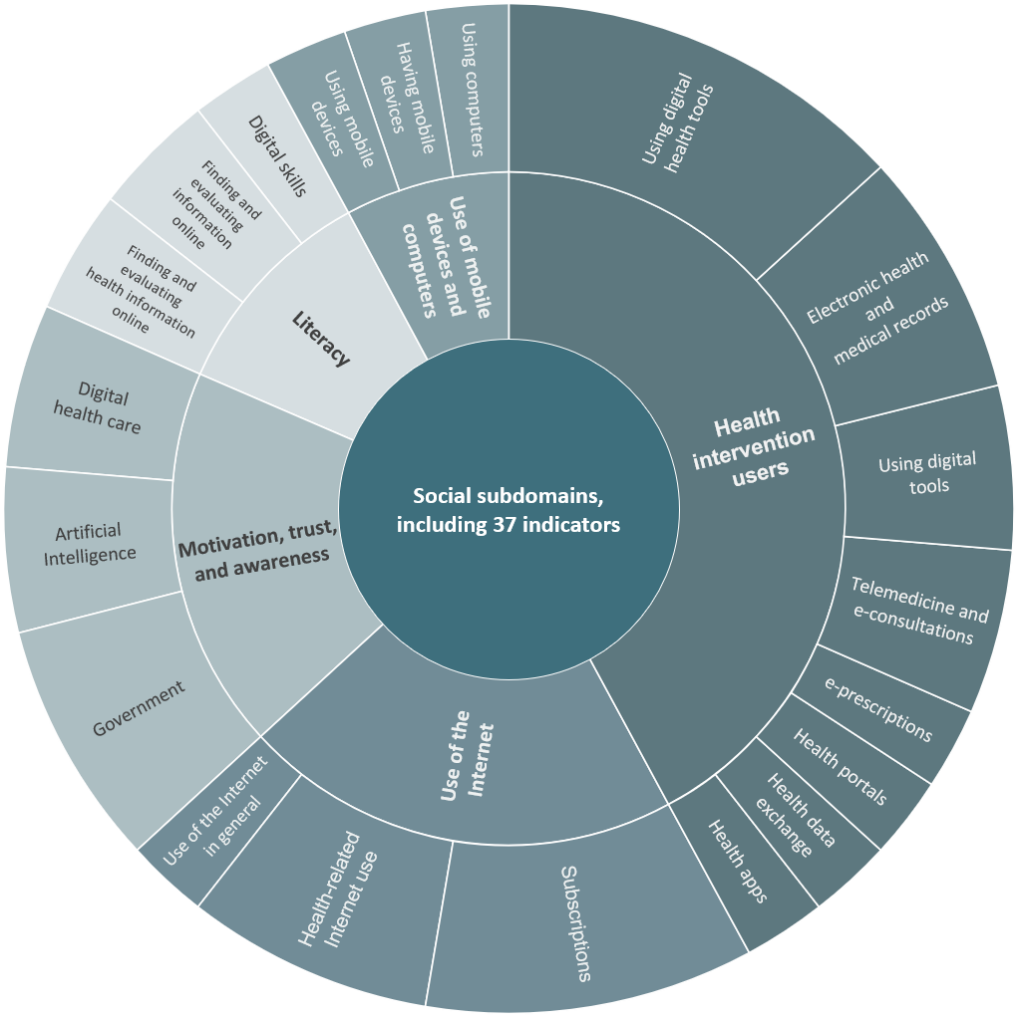
Social subdomains, including indicators per indicator clusters.

We found the least number of unique indicators tracking DiPH systems maturity and readiness related to social willingness and capability to use DiPH tools, especially in health care (37/286, 12.9%). Most indicators were related to people’s ability and capacity to use digital tools such as electronic medical records, e-prescriptions, telemedicine, health apps, and health portals (Figure 2). We found indicators tracking trust, awareness, and motivation to use DiPH tools among various populations. Indicators tracking trust explored trust concerning DiPH systems and the trust of governments and public entities supporting the development and deployment of these systems [27,68,81]. We also found indicators tracking peoples’ use of devices and digital and internet services beyond the health care setting [56,57,61,68,128,137] as well as digital literacy [124,128,131,133,138,140], identifying these as social issues with public health implications.

#### The ICT Dimension of DiPH Maturity

**Figure 3:**
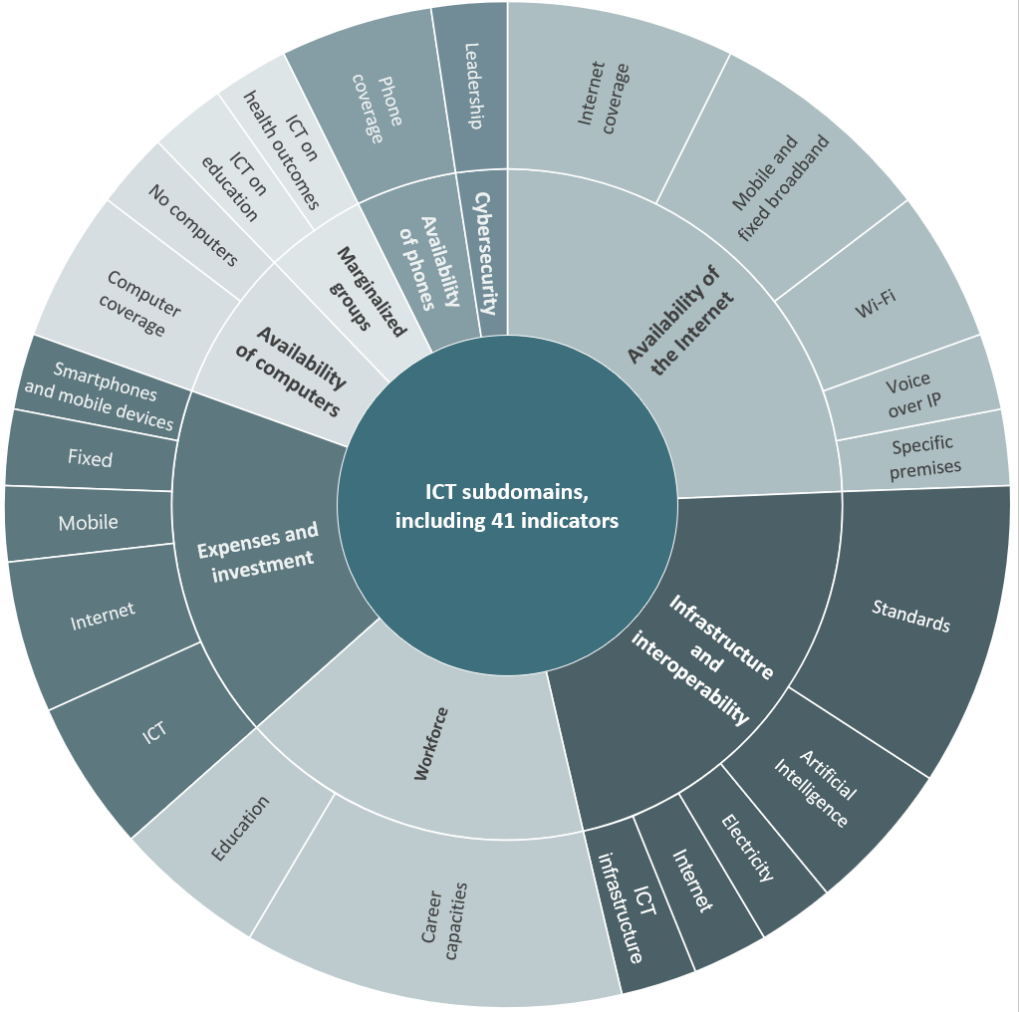
Information and communications technology (ICT) subdomains, including indicators per indicator clusters.

Overall, 41 indicators tracked ICT systems’ maturity and capacity as crucial enablers for DiPH implementation in health systems (Figure 3). Key indicators emphasized the need for the availability and accessibility of high-quality internet services [57,77,82]. They tracked the availability of mobile and fixed broadband services, the type of coverage across urban-rural divides [27,57,64,82,91,93,96,101,102,111], and the availability of public Wi-Fi infrastructure [96,100]. Similarly, indicators tracked the availability of digital infrastructure, including computers, at a population level and across different population groups [63,89,91,92,111,120]. We also found indicators assessing the existence and level of implementation of infrastructure and interoperability standards, including standards for internet services [27,70,75,106], electricity [30,95], data governance and exchange (eg, ISO standards) [27,75,106], and a general AI infrastructure (eg, computing and network capacity) [77,101]. Furthermore, we found indicators tracking investment in ICT infrastructure on a systems and population level. These include average individual or household costs of accessing internet services and acquiring digital devices alongside public or private investments in ICT infrastructure [62,105,110,114]. We also found indicators assessing the workforce’s capacity to deploy and leverage ICT infrastructure to advance public health outcomes as a part of DiPH systems maturity and readiness [26,34,64,75,76,88,90].

#### The Application of Interventions Dimension of DiPH Maturity

**Figure 4:**
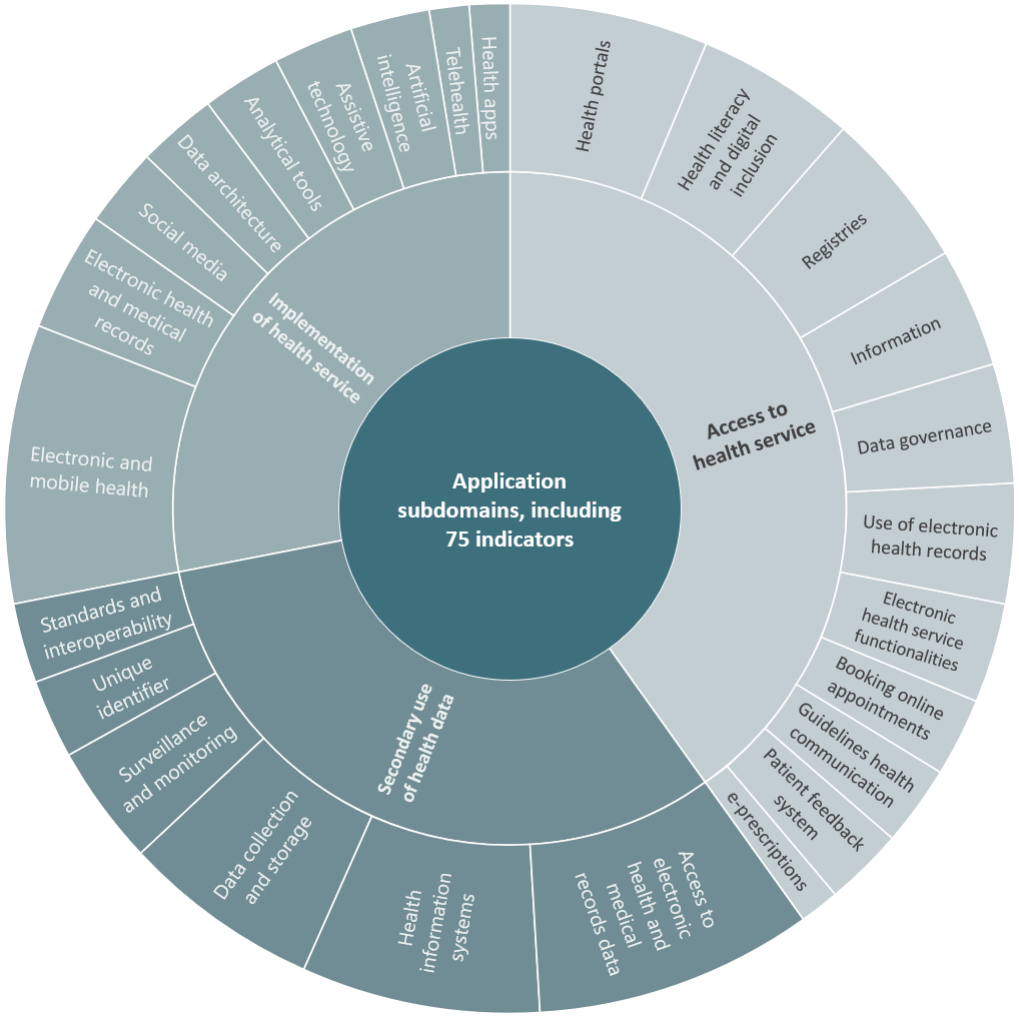
Application subdomains, including indicators per indicator clusters.

Regarding indicators tracking the development and implementation of DiPH tools, we found 75 unique indicators (Figure 4). Indicator clusters included access to DiPH services through digital tools such as health portals, online booking systems, and electronic health records [26,34,83]. We also found indicators tracking interventions to promote digital inclusion among historically marginalized groups [66,67]. Similar indicators assess DiPH services’ implementation of health apps, social media, AI, and other data analytical tools [26,27,56,64]. In addition, we identified indicators tracking the secondary use of health data from digitalized patient-facing systems for public health functions such as disease surveillance and monitoring [26,27,30,56,75]. This subdomain also tracked the implementation of interoperable health information systems and unique identifiers to allow data linkage across various public health systems to inform analyses and actions [30,64,68,76].

#### The Legal Dimension of DiPH Maturity

**Figure 5:**
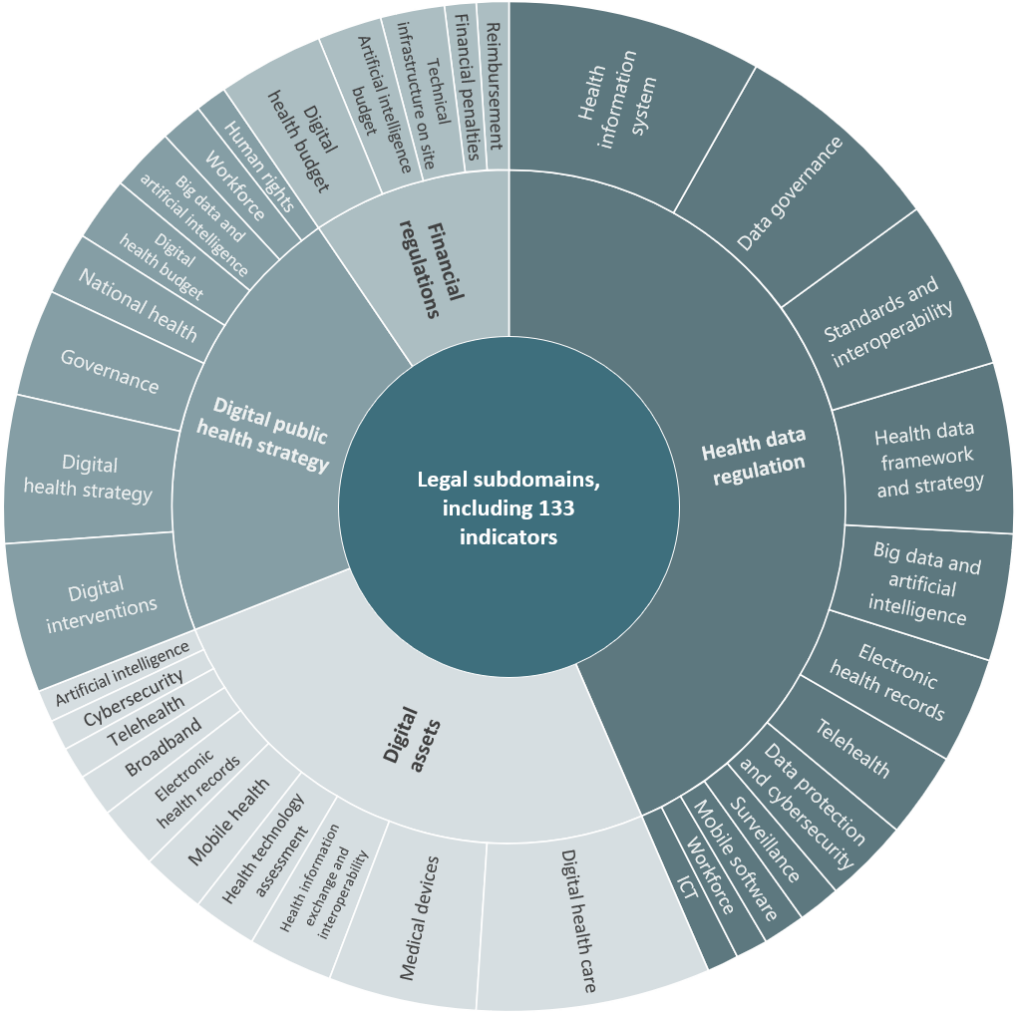
Legal subdomains, including indicators per indicator clusters.

Indicators tracking the legal and data protection regulations supporting the delivery of digital interventions dominate the literature (Figure 5). As only 4 indicators from 2 references explicitly mentioned public health [27,30], it becomes evident that most of the identified 133 legal indicators apply to digital health as the clinical subdimension of DiPH. Subdomains tracked health data regulation, including health data frameworks and strategies [26,30,59,64,70,75-77], ICT and data standards and interoperability regulations [30,64,75], data protection, governance, and cybersecurity [27,30,77]. Indicators in this domain also relate to regulating digital assets for public health, such as medical devices [30,75], health information exchange [26,30,75], health apps and portals [26,63,74-76], and cybersecurity [57]. In addition, we found indicators tracking the existence of robust digital health and DiPH strategies [58,66,68], digital governance [34,64], big data and AI [30,75], and the public health workforce capacity to leverage these DiPH tools effectively [30,66].

## Discussion

### Principal Findings

In this comprehensive review of existing indicators assessing the maturity and readiness of DiPH systems, we identified and described 286 unique indicators across 4 main domains. Among these were 37 social, 41 ICT, 75 application, and 133 legal indicators describing potential DiPH systems maturity and readiness to leverage digital systems to advance public health goals. Unsurprisingly, the largest group of indicators (133/286, 46.5%) was related to legal and data protection regulations, highlighting long-standing and potentially escalating concerns about the need for legal and regulatory frameworks to support effective development and deployment of DiPH interventions that can potentially support health for all ethically and responsibly [18].

This review extends the literature by comprehensively exploring the gray literature to outline potential indicators across 4 domains that could contribute to a holistic understanding of DiPH systems maturity and readiness. Findings from this review specifically complement those from a recent multidisciplinary Delphi study conducted by our team [15], which identified 96 indicators necessary to measure DiPH systems’ maturity. In the Delphi study, the proposed indicators overlapped with 48% (46/96) of the indicators found in the published literature [15]. While experts’ suggestions for DiPH indicators during the Delphi study implied the need for new DiPH indicators, this narrative review identified a substantial number of developed and validated indicators applicable across various digital health and DiPH contexts. Nevertheless, the question remains about how far the identified indicators and those proposed during the Delphi study will be applicable for holistically assessing DiPH systems maturity and readiness for various countries.

Currently, many jurisdictions have undertaken digital health strategies or are creating such strategies to leverage DiPH’s opportunities to optimize public health outcomes adequately [9,141,142]. However, most of these strategies have not sufficiently considered perspectives unique to DiPH; instead, they have taken a clinically and health care–focused digital health perspective [16]. Findings from our narrative review extend the literature by having more application-oriented indicators tracking the implementation of potential DiPH interventions, including leveraging electronic medical records to inform digital disease surveillance [27,56,79] using modern analytic techniques such as AI as a core of public health systems [69,76,77], and having the capacity to leverage these tools for analyses and modern communication [74,88].

### Importance of Indicators in Different Economic and Normative Settings

While the identified and selected indicators bear importance globally, one must recognize that not every indicator will have the same importance for every country. For instance, resource-poor countries with less developed electricity systems and unreliable supply will most likely see a higher relevance in the indicators targeting predictable electricity supply [143] compared with high-income countries where a reliable energy supply system has already been established and interest has shifted to setting up other systems (eg, the percentage of health care facilities and private households by access to any source of electricity [30,95]).

However, the infrastructural development of a country is not the only domain influencing the importance of indicators for a society. Cultural aspects, such as underlying normative and ethical frameworks within societies, will also play an essential role, for instance, when regulating DiPH interventions and the secondary use of health data. Kantian (individualism) and Confucian (collectivism) principles strongly influence cultures globally. In terms of DiPH, Kantian and individualism (primarily present in European and North American countries) call for active participation in managing one’s own health data, patient autonomy, and providing written consent into health data sharing and using for secondary purposes [144,145]. As such, these countries will prioritize indicators that measure the existence of regulations on sufficient health data privacy and the safety of individuals through mandatory anonymization processes (eg, the extent to which there are protections for user privacy and anonymity) [57,146].

On the contrary, countries following Confucian and collectivism principles (found predominantly in East Asia) will prioritize the public’s needs above those of individuals [147]. Following this normative framework, collectivism approaches are less likely to rate the right to manage who can access one’s electronic health record as important for the population. This is so because treatment in these societies is usually planned and executed by the health care providers alone without granting autonomy to the individual patient [148,149]. As such, indicators asking for the involvement of individuals in their health care might be less critical for countries where the principle of collectivism is followed (eg, the existence of a national legal framework for data security and privacy that addresses citizens to specify which health-related data can be shared with health professionals [26]).

Consequently, local health data governance policies can reflect local norms and values that are contrary to the indicators. These norms must be considered in the selected indicators’ answer scales and weighting procedures. Organizations and researchers must recognize cultural differences when developing indicators to not bias the results based on their own lived social norms and values.

### Comparing the Included Indicators With the GDHM Indicators as the Current Gold Standard

Mapping the collected indicators against the 31 indicators included in the GDHM reveals an overlap and also the extension of the GDHM tool through our research. While we deemed all of these indicators as relevant for our indicator list, we have also included more indicators from other sources to broaden the scope of our list compared with the GDHM. As such, our indicator list is capable of assessing the heterogeneity of topics addressed by public health and DiPH on domains where the GDHM does not.

To provide a few examples, in its first dimension, leadership and governance, the GDHM asks for governmental authorities to provide a national digital health budget; to include digital health in the national health strategy; to consider public health approaches through digital transformation; to implement emerging technologies to support public health; and to consider equity, human rights, and gender aspects in national digital health strategies [34]. For these topics, we have included more indicators on female digital skills training plans [80] to measure efforts in closing gender gaps. In addition, our indicator list includes reimbursement strategies for patients buying regulated health technologies to improve their health [26]. As another example, the GDHM dimension infrastructure requests users to assess their network readiness via the Portulans Institute’s Network Readiness Index [150]. For our list, we have excluded all indicators that would require users to evaluate topics using different tools. Instead, we have included the network readiness indicators and those from other measures in our indicator list to provide original indicators on all topics of relevance for DiPHSMR.

However, the most considerable expansions that our indicator list offers compared with the GDHM are in the social domain (for instance, asking for literacy rates among the general population and workforce [130,133]), the ICT domain (eg, on the distribution and use of computers and mobile devices among the population and health care facilities to access DiPH interventions [30,63,120]), and the use of health technologies for other public health domains such as surveillance [27,79], health promotion, or emergency response [64]. This decision followed our understanding that interventions need to be operated in a regulated and safe environment with sufficient technological infrastructure and by a knowledgeable and interested society to achieve their full potential.

### Should We Measure the Systems’ Maturity or Readiness?

The differentiated maturity and readiness assessment perspective has several implications for the identified indicators. Nevertheless, both are crucial for successfully implementing and advancing DiPH initiatives. Some of the identified indicators are more applicable for measuring DiPH maturity, focusing on the current state and sophistication of the existing system. However, others relate to DiPH readiness, which explores the system’s potential and preparedness to adopt and use emerging systems. For instance, the percentage of households with a computer [110] is a classical indicator to measure the current degree of digitalization and, therefore, the system’s maturity. However, indicators such as the percentage of patients and physicians who are comfortable with AI being used as a tool in health care [73] assess perspectives needed for the continuous development of DiPH systems (readiness assessment) as AI will play a more central role in health promotion, treatment, and surveillance in the following decades [151,152].

In addition, some indicators might be applicable for both constructs. For instance, the percentage of the population who have achieved at least a minimum level of proficiency in digital literacy skills [138] can describe the maturity of a current system as digital skills are needed to operate digital tools. However, the higher this percentage is and the higher the digital skills are, the more prepared a population will be to use emerging DiPH technologies.

While both concepts are theoretically distinct from each other, separating them in practice for assessments becomes challenging due to the fast-moving pace of the evolution of digital technologies in all sectors [9]. System evaluations such as these take their strength from the ability to benchmark not only between systems but also for the same system across multiple years. Continuously measuring the readiness of a system requires regular updates in the evaluation methodology, limiting the comparability of results over years [80].

### Strengths and Limitations

This review has multiple strengths, including its multidisciplinary focus, being implemented by researchers with expertise across various fields of public health, including clinical public health, health systems and services research, and health informatics. To further ensure the comprehensiveness of the review, our search was conducted across multiple languages (English, German, Portuguese, and Spanish) instead of being restricted to 1 or 2 languages, as is commonly done. Furthermore, this review leveraged a comprehensive approach to searching the gray literature, given the indicators are not well captured or published in peer-reviewed journals. Finally, several indicators were applied to multiple assessment tools, which increased our confidence in their importance.

However, we acknowledge the limitations inherent in the review. For example, using search engines such as Google or DuckDuckGo limited the adoption of a complex search string and a systematic search. Although DuckDuckGo claimed to display the same results to every individual due to them not collecting personal data (unlike Google), which was proven by our test for the same results as explained in the Methods section, there is still no guarantee that every person would have seen the same results as we did (known as the “filter bubble”). In addition, based on the ever-changing nature of the files and documents available on the internet, lacking archives, and the rapidly changing website domains and websites, there is a risk that the documents that were available during our screening might have disappeared or will eventually disappear [33]. This limits the reproducibility of our search and limits a review update. We are publishing this narrative review with our initially identified search results in Multimedia Appendix 1 to increase transparency. Furthermore, screening all results retrieved from Google or DuckDuckGo was impossible. Therefore, we followed the assessment process suggested by Godin et al [33], encouraging researchers to rely on the relevancy ranking in search engines and trust that the most important results will be displayed on the first pages. Unfortunately, DuckDuckGo does not display the total number of results per search (contrary to Google).

The assessment also includes a variety of limitations. Due to the broad nature of DiPH and its assessment, we identified too many indicators for a feasible content analysis. In addition, we did not statistically test for indicators measuring the same construct. Furthermore, the list of agreed-upon indicators needs to be validated by other researchers through an international and multidisciplinary Delphi study, such as the one we have previously conducted for new DiPH indicators [15].

### Further Research

This narrative review can only serve as a first step to setting ground for this relevant topic. The list of agreed-upon indicators by the authors needs to be validated by other researchers in the form of an international and multidisciplinary Delphi study, such as the one we have previously conducted for new DiPH indicators [15]. Combining both studies will result in a list of essential indicators for mapping maturity and readiness across DiPH systems. Furthermore, statistical assessments must be conducted in case studies to identify indicators measuring the same construct. They must also be tested on various DiPH systems to assess their applicability to different settings. This procedure will most likely decrease the number of indicators and make their application in evaluation procedures more feasible.

Acknowledging that academic disciplines are increasingly becoming connected leads to interdisciplinary research and essential publications outside the classic peer-reviewed articles published in scholarly journals. Instead, research is increasingly published in other formats and is not necessarily listed in major scientific databases. We encourage researchers to conduct methodological research on how to incorporate gray literature in research projects and how to systematically assess this kind of literature to increase the study results’ validity of such exercises.

In addition, future research and public health policy making must strive to prioritize the development and dissemination of well-defined, transparent indicators. These should include detailed descriptions and data sources to enhance their utility and reliability. Our study identified that only a minority of all indicators provided a definition and even fewer published their data sources (Multimedia Appendix 1, page 7). Establishing standardized guidelines for creating and reporting these indicators will not only improve their applicability but also foster greater consistency and comparability across different regions and studies. Such development is urgently needed for an impact on the evaluation of maturity and readiness of DiPH systems.

### Conclusions

This narrative review aimed to explore and consolidate the various indicators used to measure national DiPH system maturity and readiness. Through our extensive analysis, we identified 286 individual indicators that assess system maturity and readiness from multidisciplinary perspectives on topics such as digital literacy, adoption of DiPH interventions, data protection, system interoperability, investment in and regulation of DiPH interventions, and the necessary hardware and software infrastructure needed to use such tools. Furthermore, our findings reveal a critical interdependence between readiness and maturity assessments: readiness evaluations cannot be effectively conducted without first understanding the maturity of the system. Hereby, maturity assessments provide valuable insights into a system’s current capabilities. A significant issue uncovered during our review is the lack of comprehensive descriptions and data sources for the vast majority of indicators. This deficiency hampers the applicability and transparency of these indicators, ultimately limiting their usefulness for policy makers and public health researchers who rely on clear, detailed metrics to guide their decisions and strategies. As digital technologies continue to evolve, it is imperative that our methods for assessing DiPH systems keep pace, ensuring that we can accurately measure and enhance our preparedness for future challenges. By addressing these gaps and improving the robustness of our assessment tools, we can better support the advancement of DiPH initiatives worldwide, ultimately leading to more resilient and effective health systems. Finally, integrating readiness assessments with maturity evaluations will provide a more holistic view of DiPH systems, enabling more effective planning and implementation of interventions.

## Appendix

Multimedia Appendix 1 Overview of the included indicators for all 4 domains; the search strategy with results; included references; and the raw list of all extracted indicators.

## Abbreviations

AI: artificial intelligence
DiPH: digital public health
DiPHSMR: DiPH systems maturity and readiness
GDHM: Global Digital Health Monitor
ICT: information and communications technology

## Data Availability

The datasets used and analyzed during this study are available from the corresponding author upon reasonable request.
